# Effects of SARS‐CoV‐2 infection and COVID‐19 pandemic on menstrual health of women: A systematic review

**DOI:** 10.1002/hsr2.881

**Published:** 2022-10-08

**Authors:** Syeda Tayyaba Rehan, Laiba Imran, Hussain Mansoor, Qudsia Sayyeda, Hassan ul Hussain, Mustafa Sajjad Cheema, Muhammad Junaid Tahir, Muhammad Sohaib Asghar, Mohammed Mahmmoud Fadelallah Eljack, Md. Saiful Islam

**Affiliations:** ^1^ Department of Medicine Dow University of Health Sciences Karachi Pakistan; ^2^ Department of Medicine Red Crescent of Tampa Bay Tampa Florida USA; ^3^ Department of Medicine CMH Lahore Medical College Lahore Pakistan; ^4^ Department of Medicine Lahore General Hospital Lahore Pakistan; ^5^ Department of Internal Medicine Dow University of Health Sciences–Ojha Campus Karachi Pakistan; ^6^ Division of Nephrology and Hypertension Mayo Clinic Rochester Minnesota USA; ^7^ Department of Community Medicine, Faculty of Medicine and Health Sciences University of Bakht Alruda Ad Duwaym White Nile state Sudan; ^8^ Department of Public Health and Informatics Jahangirnagar University Savar Dhaka Bangladesh; ^9^ Centre for Advanced Research Excellence in Public Health Savar Dhaka Bangladesh

**Keywords:** COVID‐19, females, menses, psychological impact, quality of life, stress

## Abstract

**Background:**

The menstrual cycle in women is the main indicator of their reproductive health which is affected by the ongoing coronavirus disease 2019 (COVID‐19) pandemic. This review aims to summarize the effects of the COVID‐19 infection and the global pandemic on the menstrual health of women.

**Methods:**

The literature search was conducted in PubMed, Cochrane library, and Google Scholar using keywords “COVID‐19,” “Menstrual Cycle,” “Menstrual Cycle Irregularities,” “Amenorrhea,” “Polymenorrhea,” and “Dysmenorrhea.” The articles were selected according to the following inclusion criteria: (i) cross‐sectional studies, (ii) cohort studies, (iii) surveys, and (iv) other observational studies observing the effects of SARS‐CoV‐2 infection or COVID‐19 pandemic on menstrual health of women. Exclusion criteria included: case reports, gray literature, and website articles regarding menstrual health.

**Results:**

A total of 30,510 articles were shortlisted after a comprehensive search. Sixteen articles were included out of which 13 studies investigated the effects of the COVID‐19 pandemic on the menstrual cycle while 3 evaluated the possible effects of COVID‐19 infection on the menstrual health of women. Menstrual disorders or irregularities were a more common finding during the pandemic as compared to before (*p* = 0.008). Women affected by pandemic‐related stress were more prone to changes in the duration of their menses (*p* = 0.0008), reported heavier bleeding (*p* = 0.028), and increased incidence of painful periods (*p* < 0.0001). COVID‐19 infected women also reported changes in their menstrual cycle including irregular menstruation, increased symptoms of premenstrual syndrome, and infrequent menstruation.

**Conclusions:**

Women suffering from COVID‐19 infection or pandemic‐associated stress and anxiety were more likely to experience irregular menstruation, dysmenorrhea, amenorrhea, and other menstrual abnormalities compared to those who were less exposed.

## 1 INTRODUCTION

The ongoing coronavirus disease 2019 (COVID‐19) pandemic has not only created burdens on the healthcare system but also led to the disruption of the social structure of societies. While the pandemic has taken its toll on almost everyone, women have had to deal with more profound challenges, both at the workplace and at home. During the pandemic, chronic symptoms of psychological distress such as anxiety and stress have become rampant among populations across the globe.^1^ A previous study showed that a significant increase in the anxiety of women was reported as the State‐Trait Anxiety Inventory scores‐I (STAI‐I) during the course of the pandemic as compared to the State‐Trait Anxiety Inventory scores‐II (STAI‐II) that highlighted the general anxiety of the participants.^1^

Amidst the peak of the coronavirus outbreak in 2020, CARE International conducted a report inculcating the first‐person accounts of more than 10,000 participants regarding the challenges they faced during the COVID‐19 pandemic. The results showed that 27% of the female participants reported an increase in mental health issues compared to only 10% of the males.^2^ Exactly a year later in 2021, CARE's rapid gender analysis based on the impact of COVID‐19 on the daily lives of individuals revealed that women were three times more prone to experiencing adverse mental health struggles, as compared to men. This disparity can somewhat be attributed to the widely accepted notion that women are more susceptible to mental health troubles, owing to their more anxious temperament.^3^ Due to this trait‐like phenotype, women are naturally at a higher risk of experiencing mental health problems such as chronic anxiety, stress, depression, sleep disturbances, and posttraumatic stress disorder.^4^ The pandemic has not only exacerbated mental health issues within the female population but led to poor outcomes.

Following the coronavirus outbreak in 2020, numerous reports from various countries highlighted a significant increase in cases of domestic violence against women.^5^ Due to the lockdown, people were forced to stay indoors and this, unfortunately, resulted in more women being subjected to physical and mental abuse within their homes. According to a Jordanian cross‐sectional study, 20.5% of females reported that they suffered from domestic abuse during the COVID‐19 pandemic.^6^ This can be attributed to causative factors such as financial insecurity, lack of social support, quarantine, unstable relationships, and limited healthcare and domestic violence support options.

The menstrual cycle is an integral regulator of the female reproductive function and is highly susceptible to psychological disruptions such as stress, depression, and insomnia.^7^, ^8^ Various factors like menstrual frequency, the amount of menstrual bleeding, and the length of bleeding have an intimate correlation with the type of psychological stressors affecting the individual. Pre‐COVID‐19 findings highlighted that acute stress affects the regions of the brain that regulate emotions during the luteal phase of the cycle, in contrast to the findings in the late follicular phase. It was observed that increased sympathetic activity during stages of acute stress has a trickle‐down effect on different areas of the brain, particularly the amygdala which is associated with strong emotions like pleasure and fear. This increase in neural activity of the brain, especially due to psychological distress during the late luteal phase, causes disruptions in the normal pregnenolone levels of the body, leading to grave menstrual complications.^9^

A prior study conducted among Korean women determined a correlation between mental health and the menstrual cycle. The findings also disclosed that poor mental health status leads to irregularities in the menstrual cycle of women.^10^ These were also validated by the findings of an observational study that assessed the changes in women's reproductive health following the COVID‐19 outbreak. The statistics from this study show that there were significant changes in the length of menstruation in women who had reported their quality of life is adversely affected by the pandemic.^11^

There are several factors associated with the COVID‐19 pandemic that have contributed to triggering such mental health challenges in women. Social isolation coupled with an unhealthy household environment during the quarantine periods proved to be detrimental to the mental well‐being of women. There was also a strong correlation between episodic depression and unemployment among women who lost their jobs during the pandemic.^12^ Furthermore, the fear of infection and related mortality proved to be another major risk factor for the deteriorating mental health of women during the pandemic.

In a nutshell, COVID‐19 is not just a respiratory illness, but has several adverse effects throughout the body, especially in women. COVID‐19 infection along with the associated stress of the COVID‐19 pandemic has had a serious impact on the reproductive health of women leading to a change in the duration of menstrual cycles.^11^ The present review reports aimed to discuss the changes caused by COVID‐19 infection and its pandemic on the menstrual health of women.

## 2 METHODS

This review was carried out following the Preferred Reporting Items for Systematic Reviews and Meta‐Analyses (PRISMA) 2020 guidelines (Supporting Information: File 1). This review is registered on PROSPERO (Registration number: CRD42022332805).

### 2.1 Search strategy and data sources

A comprehensive search was performed in PubMed, Cochrane Library, ERIC (Education Resources and Information Center), and Google Scholar from their inception to May 19, 2022, by using the following search terms “(COVID‐19 OR SARS‐CoV‐2 OR coronavirus) AND (Menstrual Cycle OR Irregular Menstrual Cycle OR Menstrual Cycle Irregularities OR Delayed Menstrual Cycle OR Early Menstrual Cycle OR Prolonged Menstrual Cycle OR Menstrual Cycle Disturbances OR Amenorrhea OR Polymenorrhea OR Dysmenorrhea OR Hypomenorrhea OR Hypermenorrhea).” No filters were applied during the literature search over any of the search engines. The search strategy was slightly adapted for each database and a detailed search string for each database is presented in Supporting Information: File 2.

### 2.2 Study selection

Two reviewers independently screened titles and abstracts for eligibility. All the articles that addressed the menstrual experience of severe acute respiratory syndrome coronavirus‐2 (SARS‐CoV‐2) infected and noninfected women during the pandemic were retained separately. Full‐text articles were then independently reviewed by the two reviewers for inclusion. Finally, discrepancies were resolved through discussion between the two reviewers, and a third reviewer if necessary.

### 2.3 Inclusion and exclusion criteria

All the cross‐sectional surveys, cohort analyses, and observational studies investigating the effects of the COVID‐19 pandemic or SARS‐CoV‐2 infection on the menstrual health of women were included in this review. Case reports, gray literature, and website articles were excluded from data extraction. Studies evaluating the effects of COVID‐19 vaccination on menstrual cycles were also excluded from this review. There was no language‐based exclusion since all the available articles were published primarily in English.

### 2.4 Population

The following two groups of the population were considered eligible for this review.

Group 1: Reproductive‐age women that were not tested for COVID‐19 infection.

Group 2: Reproductive‐age women that were confirmed COVID‐19 positive via laboratory confirmation.

Exposure: For Group 1, the exposure was the COVID‐19 pandemic and for Group 2 individuals the exposure was SARS‐CoV‐2 infection.

Comparator: (a) Studies that compared the menstrual changes of women during the COVID‐19 pandemic with their previous cycles before the pandemic were considered eligible for this review. (b) Studies that compared the menstrual changes in SARS‐CoV‐2 infected women with the cycles of noninfected women or their own cycles before infection are included in this review.

Outcomes: Menstrual irregularities (early or delayed cycle), duration of menstruation (prolonged menstrual cycle), dysmenorrhea, amenorrhea, the volume of bleeding (heavy periods), endometriosis symptoms, premenstrual syndrome symptoms were the outcomes that have been extracted from the included studies. The relation of these outcomes with anxiety and fear of the COVID‐19 pandemic and SARS‐CoV‐2 infection has been assessed separately.

### 2.5 Data extraction

Data extraction was completed by two independent reviewers, and discrepancies were resolved through feedback from a third reviewer. Data were extracted from the articles including the patient characteristics, COVID‐19 infection, anxiety or stress during the pandemic, and menstrual health factors (e.g., cycle duration, blood flow, volume, pain during the cycle, premenstrual symptoms, and irregular or missed periods during the pandemic). All data were extracted in a predesigned excel sheet. Menstrual experiences of COVID‐19‐infected and noninfected women were retrieved in a separate sheet.

### 2.6 Data synthesis

The outcomes of recruited studies were qualitatively synthesized and not combined for meta‐analysis due to the different clinical and methodological approaches used in the studies. Study findings are summarized in Section 3 and tabulated in summary tables.

### 2.7 Quality assessment

The Newcastle Ottawa scale was used for assessing the quality of the included observational studies (Supporting Information: File 3). It was based on the selection of participants, comparability of participants, and outcome/exposure criterion of included studies.

## 3 RESULTS

**Figure 1:**
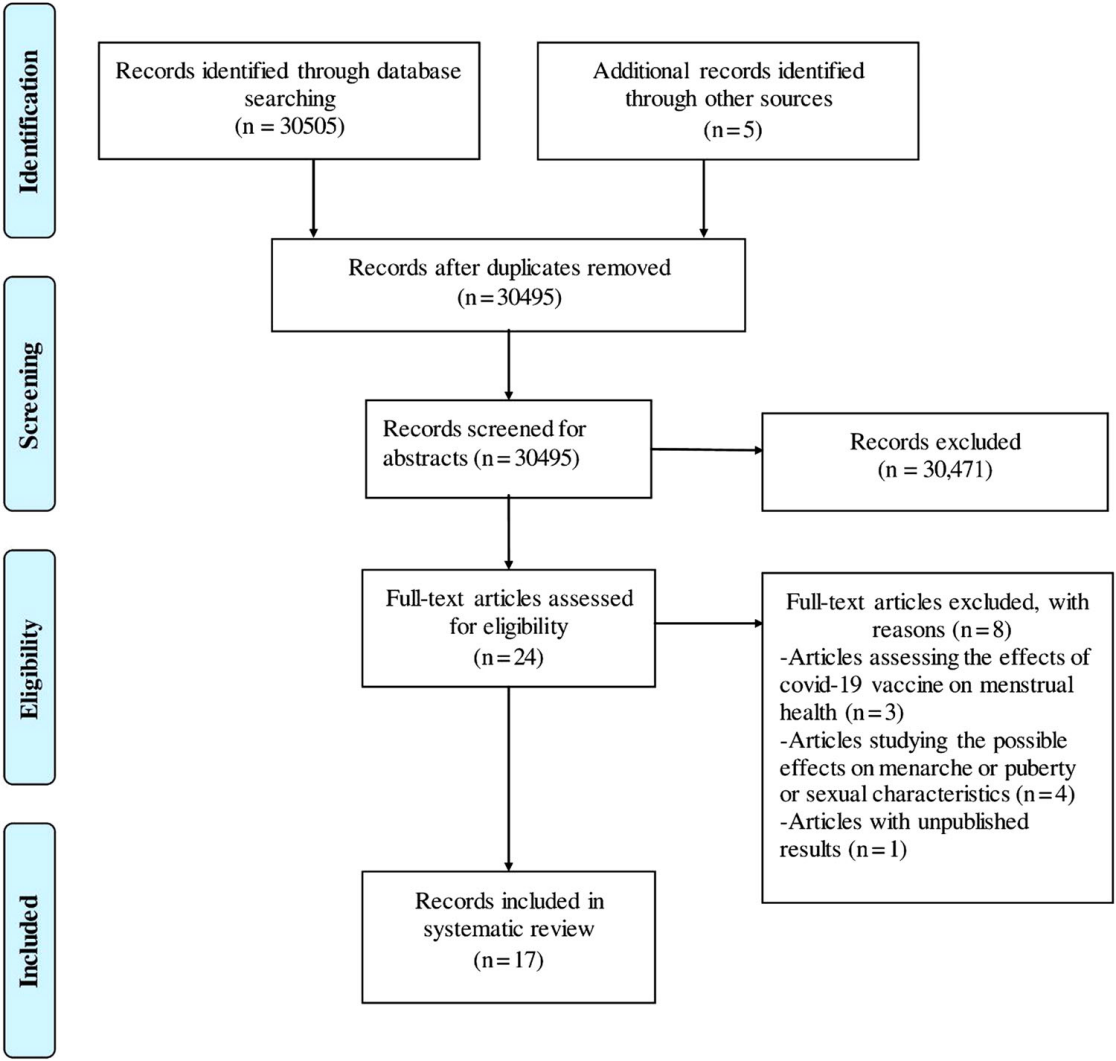
Preferred Reporting Items for Systematic Reviews and Meta‐Analyses (PRISMA) flow chart of literature search

After performing a comprehensive search on 3 electronic databases, a total of 30510 articles were recruited (Figure 1). These articles were exported to EndNote Reference Manager (Version X 7.5; Clarivate Analytics). After removing duplicate articles and abstract screening, 24 articles were shortlisted and 8 articles were further excluded after a full‐text review. Finally, 17 articles were included out of which 9 were cross‐sectional studies, 6 cohort studies, 1 survey, and 1 anonymous observational study. Of these, 13 studies investigated the effects of the COVID‐19 pandemic on the menstrual cycle (see Table 1), while 3 evaluated the possible effects of COVID‐19 infection on the menstrual health of women (see Table 2).

**Table 1:** Features of studies assessing the impact of the global pandemic‐associated stress on the reproductive health of women

References	No. of participants	Type of study	Aims of Study	Scale used to assess mental health symptoms	Mental health assessment	Outcomes
Maher et al.^18^	*N* = 1335	Cross‐sectional study	To evaluate the long‐term reproductive and mental health repercussions of the COVID‐19 pandemic	Patient Health Questionnaire‐9 (PHQ‐9), General Anxiety Disorder‐7 (GAD‐7)	Women reported worsening mental health symptoms including anxiety, stress, low mood and loneliness during the pandemic	The incidence of dysmenorrhea, heavy periods, and missed periods significantly increased during the pandemic (*p* < 0.000). Increased anxiety led to a shift from nonpainful to painful periods and worsening premenstrual symptoms.
Aolymat et al.^21^	*N* = 385	Cross‐sectional study	To evaluate the impact of the COVID‐19 pandemic on dysmenorrhea and PMS symptoms in women	Depression, anxiety, and stress scale‐21 (DASS‐21)	The depression anxiety and stress were significantly increased from during the COVID‐19 pandemic (*p* = 0.000)	COVID‐19 associated depression, anxiety, and stress scores were positively correlated with PMS components and dysmenorrhea (*p* < 0.05).
Buran and Gerçek Öter.^22^	*N* = 125	Cross‐sectional study	To assess the impact of COVID‐19 associated fear on the menstrual cycles of women	Awareness of COVID‐19 scale (ACV‐19S).	High fear of COVID‐19 scale score was observed	A significant association between women's COVID‐19 fear and menstrual symptoms was observed (*p* < 0.001).
				Fear of COVID 19 Scale (FCV‐19S)		
Ozimek et al.^20^	*N* = 210	Retrospective cohort study	To study the effects of the COVID‐19 pandemic on the menstrual cycles of women	Perceived Stress Scale (PSS)	The average covid perceived stress scores of participants were significantly higher during the pandemic (*p* < 0.0001)	A significantly positive association between high covid perceived stress scores and menstrual changes was observed (*p* < 0.0008).
Haile et al.^11^	*N* = 1159	Retrospective cohort study	To assess the changes in women's reproductive health associated with the COVID‐19 pandemic	Authors designed their own survey assessing psychological distress	66.4% (178/268) of the women reported feeling stressed because of the pandemic	Significant changes in the menstrual length of women who were stressed during the pandemic were observed (*p* = 0.0001).
Dutta et al.^17^	*N* = 231	Survey	To evaluate the effect of sleep cycles during the COVID‐19 lockdown on the menstrual health of women	Self‐made questionnaire	46.75% of the study participants were found stressed during the pandemic (*p* < 0.05)	Significant relation, between sleep duration, and irregularities in the menstrual cycle, was observed (*p* < 0.05).
Schwab et al.^23^	*N* = 285	Cross‐sectional study	The study aims to investigate the impact of the pandemic on the pain intensity of endometriosis patients	Self‐made questionnaire	21.9% of the patients reported feeling less supported by their family (*p* < 0.001)	Menstrual pain was significantly low in endometriosis patients despite feeling less supported by family and friends (*p* < 0.001).
Nguyen et al.^16^	*N* = 18,076	Retrospective cohort study	To detect the variations in ovulation and menstruation during the COVID‐19 pandemic	Self‐made questionnaire	45.4% of the participants reported more pandemic‐related stress. 33.2% reporting no changes in stress and 21.4% reported less stress compared to the prepandemic period (*p* < 0.05)	No significant association was detected between the stress scores and abnormal menstrual parameters.
Demir et al.^1^	*N* = 263	Cross‐sectional study	To examine the effect of the pandemic on the menstrual cycles of women	State Anxiety Inventory (STAI‐1 and 2)	High anxiety levels were recorded during the pandemic among study participants (*p* < 0.05)	A positive correlation between menstrual symptoms and COVID‐19 anxiety was recorded (*p* = 0.004).
Phelan et al.^14^	*N* = 1031	Anonymous observational study	To assess the impact of the COVID‐19 Pandemic on the reproductive health of women	Digital survey	Women reported a significant increase in suffering from mental health symptoms during the pandemic (*p* < 0.0001)	A positive association between compromised mental health and menstrual symptoms was reported (*p* < 0.0001).
					50% of women reported low mood (*p* < 0.0001)	
					50% of women reported anxiety (*p* < 0.0001).	
					36% women reported significant stress (*p* < 0.0001)	
Takmaz et al.^19^	*N* = 952	Cross‐sectional study	To assess the impact of COVID‐19 associated depression, and anxiety on the menstrual health of healthcare workers	COVID‐19 Stress Scales (CSS), Depression Anxiety Stress Scale (DASS‐21)	The CSS and DASS‐21 scores were significantly higher in the irregular menstruation group (*p* < 0.001)	A positive correlation was found between pandemic induced stress and menstrual abnormalities (*p* < 0.001).
Prabowo et al.^15^	*N* = 348	Cross‐sectional study	To evaluate the effects of working from home on the reproductive health of women during the pandemic	General Health Questionnaire (GHQ‐12)	Psychological distress was observed in 48% of the participants	No significant difference was found between work from home and the menstrual health of women (*p* > 0.05).
Aolymat^6^	*N* = 200	Cross‐sectional study	To assess the correlation of the COVID‐19 pandemic with various variables including menstrual health	‐	Not assessed	N/A
Yuksel and Ozgor^13^	*N* = 58	Prospective cohort study	To examine the impact of the pandemic on the sexual behavior of women	‐	Not assessed	N/A

**Table 2:** Characteristics of studies evaluating the effects of COVID‐19 infection on menstrual health of women

References	No. of participants	Type of study	Aims of study	Findings
Khan et al.^24^	*N* = 127	Prospective cohort study	To assess the changes in menstrual cycle with SARS‐CoV‐2 infection	A positive association was found between COVID‐19 infection and menstrual irregularities in women.
Li et al.^25^	*N* = 237	Cross‐sectional study	To evaluate the effects of SARS‐Cov‐2 infection on ovarian reserve, sex hormones, and menstruation in women of child‐bearing age	As compared to controls, patients infected with COVID‐19 reported changes in menstrual volume (*p* < 0.001) and menstrual cycles (*p* < 0.001).
Ding et al.^26^	*N* = 78	Cohort study	This survey is aimed to investigate the relationship between COVID‐19 disease and ovarian function in reproductive‐aged women	No significant association between COVID‐19 infection and menstrual changes (*p* > 0.05).

Abbreviations: COVID‐19, coronavirus disease 2019; SARS‐Cov‐2, severe acute respiratory syndrome coronavirus‐2.

### 3.1 Quality assessment

Newcastle Ottawa scale was used to carry out a quality assessment of the included studies with scores ranging between 4 and 8 inclusive out of a maximum of 8 for cross‐sectional studies and between 6 and 7 inclusive out of a maximum of 9 for cohort studies as depicted in the quality assessment in Table S1 and S2 of the Supporting Information: File 3, respectively.

### 3.2 Effects of COVID‐19 pandemic on menstrual health of women

To evaluate the effects of the pandemic on the menstrual cycle of women, several studies have been designed and conducted, primarily focusing on how pandemic‐associated stress led to changes in women's menstrual cycles and to what extent the cycles were affected. Major and minor impacts of the COVID‐19 pandemic on the menstrual health of women are mentioned below.

### 3.3 Irregularities in the menstrual cycle

It was observed that menstrual disorders or irregularities were a more common finding during the pandemic as compared to before (27.6% vs. 12.1%, *p* = 0.008).^13^ A study reports that women who suffered from mood swings, anxiety, or stress were at a higher risk of experiencing changes in their menstrual cycles since the beginning of the pandemic (50% vs. 34%, *p* < 0.0001).^14^ Women who had started working from home during the pandemic also experienced menstrual irregularities compared to their menstrual cycles earlier.^15^ On the contrary, one study showed that women reported a decrease in menstrual abnormalities amidst the curfew imposed during the pandemic, 10.5% during the pandemic versus 17.5% before it.^6^ Data from another study show that the incidence of abnormal cycle length and anovulatory cycles decreased during the pandemic from 8.7% to 8.0% and 2.9% to 2.5%, respectively. Participants over the age of 45 were more prone to experiencing menstrual aberrations like anovulatory cycles and abnormal cycle length.^16^ While stress and anxiety associated with the pandemic were found to be the major factors impacting the menstrual cycles of women, a study showed that women with menstrual irregularities experienced significant changes and fluctuations (*p* < 0.01) in their bedtime during the pandemic.^17^ Maher et al.'s^18^ study found that poor sleep was a significant independent predictor of missed periods (odds ratio [OR] = 1.11, 95% confidence interval [CI] = 1.03–1.19) and overall menstrual cycle disruption (OR = 1.11, 95% CI = 1.05–1.18).

### 3.4 Duration of the menstrual cycle

Fifty‐six percent of women enrolled in a study reported experiencing a difference in their menstrual cycle since the start of the pandemic with a greater variability seen in cycle length (18). A study conducted by Demir et al. showed that there was a decrease in the number of pads used by women aged 18–45 during their menstrual cycle amidst the pandemic even though these women reported experiencing regular menstruation before the pandemic (3.7 ± 2.6 pads/day before pandemic vs. 3.2 ± 1.5 pads/day during a pandemic). The duration of the menstrual cycle also decreased among these women (period time 6.3 ± 2.1 [prior pandemic] vs. 5.9 ± 1.8 [during pandemic]). These changes may be attributed to the high STAI‐I scores observed in the study which are a benchmark of anxiety scores during the course of the pandemic.^1^ Similar findings from Nguyen et al. show that on average, the length of the menstrual cycles of the participants decreased from 29.40 days before the pandemic to 29.16 days during the course of the pandemic (*p* < 0.001). The incidence of longer menstrual cycles went from 0.9% to 0.1%. The aforementioned irregularities may correlate to the fact that the percentage of participants who were extremely stressed increased during the COVID‐19 pandemic from 46.2% before the pandemic to 61.1% during the pandemic.^16^

The previously mentioned results are in contrast with the findings of Haile et al.^11^ who reported that women seriously affected by the pandemic reported an increase in the duration of their menstrual cycle. Out of the 269 participants, 119 (44.4%) stated that they had observed aberrations in their menstrual cycle for the past 12 months The incidence of this finding was slightly higher in women who were affected by the pandemic or had a family that was affected as compared to women who were not (53.9% vs. 46.1%).^11^

A study evaluating the effect of the pandemic on the menstrual cycle of healthcare providers reports that out of 659 participants included in the study, 10.7% of women reported an increase or decrease in the length of their menstrual cycle and 6.5% of the participants reported intermenstrual bleeding. These irregularities can be explained by the significantly high Cyberchondria Severity Scale (CSS) scores in women experiencing irregular menstruation (*p* < 0.001). Similarly, the depression, anxiety, and stress, subdimensions of the Depression, Anxiety, and Stress Scale‐21 (DASS‐21), were found to be significantly higher in women in the irregular menstruation group (*p* < 0.001).^19^ Important findings from Ozimek et al. show that out of 210 total participants, 54% reported changes in their menstrual cycle during the pandemic including duration of menses (34%) and schedule of the cycle (50%). It was observed that participants with high COVID‐19 Perceived Stress Scale (PSS) scores were more prone to changes in the duration of their menses compared to those with relatively lower scores (58% vs. 29%, *p* = 0.0008).^20^

### 3.5 Heavy periods

Out of the 1031 participants included in a study by Phelan et al.,^14^ 47% of women experienced heavy periods, and 5% reported that the incidence had increased during the pandemic (*p* = 0.003). Similarly, Maher et al.'s^18^ study also demonstrated a significant increase in heavy menstrual bleeding compared to prepandemic bleeding (*p* < 0.0001). Data from Ozimek et al. (*n* = 210) showed that women with high COVID‐19 PSS scores were more prone to experiencing changes in their menstrual bleeding pattern during the pandemic as compared to women with moderate PSS scores (71% vs. 50%, *p* = 0.020). Similarly, women displaying higher scores were also more likely to report heavier bleeding compared to those with lower scores (42% vs. 24%, *p* = 0.028).^20^

### 3.6 Dysmenorrhea

According to a study conducted by Phelan et al.,^14^ 49% of the women reported painful periods and 7% stated that the incidence of painful periods had increased during the pandemic (*p* < 0.0001). For 30% of the women, painful periods were a new occurrence while 12% of the women who experienced painful periods before the pandemic, reported that the pain had improved during the pandemic. It was observed that women who struggled with their mental health were more predisposed to painful periods (54% vs. 36%, *p* < 0.0001).^14^ COVID‐19‐associated anxiety was linked to an increase in the severity of dysmenorrhea in women (*p* = 0.025) whereas depression caused by COVID‐19 was also linked with worsening dysmenorrhea (*p* = 0.008). The incidence and severity of dysmenorrhea increased during the pandemic as 49.9% of the women involved in the study experienced it as compared to 36.9% before the pandemic.^21^ This is supported by evidence from another study which shows that out of 125 participants, 22.4% experienced increased menstrual pain during the pandemic as compared to before, and 25.6% of women reported worsening menstrual cycles.^22^ Maher et al.'s^18^ study also corroborates that the change from nonpainful to painful periods was linked with anxiety during the pandemic.

Amenorrhea Women described a significant increase in missed periods during the pandemic^18^ with reports from Phelan et al. showing that 17% of women reported missed periods during the pandemic out of which for 9% of them it was a new occurrence. A total of 17%–21% reported that while they missed periods occasionally before the pandemic, the incidence of missed periods had increased during the pandemic.^14^

### 3.7 Premenstrual syndrome (PMS) symptoms

Results show that premenstrual syndrome symptoms like headache (*p* = 0.017) and palpitations (*p* = 0.000) were also linked with heightened anxiety due to the COVID‐19 pandemic^21^ and women who experienced mental health issues were prone to deteriorating premenstrual symptoms (62% vs. 32%, *p* < 0.0001).^14^ Further evidence shows that 50% of the participants included in Ozimek et al.^20^ reported changes in premenstrual symptoms during the course of the pandemic. Similarly, out of the 1335 women included in Maher et al.'s^18^ study, 64% reported deteriorating premenstrual symptoms which were directly linked with increased anxiety during the pandemic.

### 3.8 Endometriosis

Schwab et al. evaluating the effect of the pandemic on patients with endometriosis showed that out of the 285 participants included in the study, 15.9% (*p* = 0.31) reported increased consumption of over‐the‐counter pain medication while 15.9% of the participants reported increased consumption of prescribed pain medication (*p* = 0.91). The perception of pain in these patients also changed significantly during quarantine as 29.3% of the patients reported that they were more worried about their pain (*p* = 0.02) and 43.6% (*p* < 0.001) reported heightened awareness of their pain.^23^

### 3.9 Effects of SARS‐CoV‐2 infection on menstrual health of women

According to a CoVHORT analysis conducted in Arizona state, 16% of the COVID‐19‐infected women reported changes in their menstrual cycle. The most commonly reported changes in these participants were irregular menstruation (60.0%), an increase in premenstrual syndrome symptoms (45.0%), and infrequent menstruation (35.0%).^24^

A study by Li et al. observed that 132 out of 177 COVID‐19‐positive participants noticed no change in their menstrual volume. On the other hand, 36 patients had a significant decrease in menstrual volume and 9 patients reported an increase in volume.^25^ To evaluate the association between the disease's severity and its effects on the menstrual cycle, the authors further compared the menstrual cycles of women who were mildly infected with coronavirus and the women who were severely ill with the disease. It was found that 88 out of 147 mildly ill patients reported no changes in menstrual volume, while 24 had decreased and 7 had increased menstrual volumes. In a group of severely ill patients, 44 out of 90 women reported no change in their menstrual volume, 12 participants had decreased while 2 of them had increased volumes. Twenty‐one mildly ill patients and 12 severely ill patients showed prolonged menstrual cycles. Twenty‐three mildly ill patients had cycles longer than 37 days while in the severely ill group, 20 patients had cycles longer than 37 days. Prolonged menstrual cycles were more commonly reported in comparison to short‐length cycles.^25^

An observational study by Ding et al. reported that 15% of the women infected with COVID‐19 had experienced irregular menstrual cycles or missed periods. Authors indicated that no obvious menstrual cycle change was observed, however, women affected by COVID‐19 have significantly lower serum anti‐Mullerian hormone levels and higher serum testosterone and prolactin levels. This abnormal reproductive hormone fluctuation suggests a poor ovarian reserve when compared to healthy unaffected women of the same age.^26^

## 4 DISCUSSION

Our systematic review is among the few studies conducted that summarize the effects of COVID‐19 infection and its pandemic on the menstrual health of women. The COVID‐19 pandemic brought difficulties and challenges for humankind while leaving the mental health of women in a vulnerable state. Mental illness not only compromises cognitive ability but also hinders the physical well‐being of women. Reproductive system of women is discerned to be on the verge of getting affected by mental disability due to the abnormal fluctuations in the menstrual cycle's regulatory and inhibitory hormones.^7^, ^8^, ^9^

We found that amidst the pandemic while most of the women remain unaffected in terms of their menstrual cycles, the ones who were suffering from anxiety, stress, and depression reported changes in their menstrual patterns.^14^, ^15^, ^16^, ^17^ Major changes that were recorded include increased or decreased menstrual volume, prolonged duration of the cycle, increased episodes of pain, and occurrence of PMS symptoms.^16^, ^17^, ^19^, ^20^, ^21^, ^22^ The participants who were already suffering from endometriosis reported a significant increase in pain frequency during the pandemic time period.^23^

The SARS‐CoV‐2 infection itself has proven to be a systemic disorder. Studies disclose the effects of the lethal virus on the reproductive health of women that include dysregulation in several regulatory and inhibitory reproductive hormones e.g., prolactin, AMH, and testosterone.^26^

The regular menstrual cycle assures the maintenance of the uterus through its periodic shedding and regeneration. Irregularities in the menstrual cycle can pave the path for certain complications including pre‐eclampsia, low birth weight of the fetus, spontaneous delivery, and increased risk of metabolic disorders.^27^, ^28^ Factors contributing to psychological illness should be analyzed and discussed to break the chain of poor mental effects on the reproductive activity of women.

### 4.1 Limitations

The long‐term impact of COVID‐19 infection and subsequent pandemic on menstrual health had not been studied in any of the included surveys. This review did not address the effects of the COVID‐19 vaccine on women's reproductive health.

### 4.2 Strengths

The review encourages researchers to investigate the long‐term effects of the COVID‐19 pandemic and SARS‐CoV‐2 infection‐related menstrual irregularities in‐depth, and it calls for psychosocial committees to design preventive measures and protocols for any future crises.

## 5 CONCLUSIONS

Our review discloses a significant relation between abnormal menstruation and COVID‐19 pandemic‐associated stress, fear, and depression. Second, a positive interlink has been observed between SARS‐CoV‐2 infection and menstrual changes. Regular assessments of mental health status during times of crisis, such as a pandemic, should be maintained to explore mental illnesses in their early stages. The government should invest in preventive measures including easily accessible psychological first aid centers and mental health awareness programs for women from all socioeconomic backgrounds. Expert psychologists and stress therapists can be taken on board to design a tele‐mental health program to provide mental health services via telecommunications or video conferencing technologies. More research is needed to determine the long‐term consequences of the COVID‐19 pandemic and SARS‐CoV‐2 infection on women's menstrual cycles.

## AUTHOR CONTRIBUTIONS

*Conceptualization*: Syeda Tayyaba Rehan, Laiba Imran. *Formal analysis*: Hussain Mansoor, Qudsia Sayyeda. *Writing–original draft*: Syeda Tayyaba Rehan, Laiba Imran, Hussain Mansoor, Muhammad Junaid Tahir, Muhammad Sohaib Asgha. *Writing–review and editing*: Qudsia Sayyeda, Hassan ul Hussain, Mustafa Sajjad Cheema, Mohammed Mahmmoud Fadelallah Eljack, Md. Saiful Islam.

## CONFLICT OF INTEREST

The authors declare no conflict of interest.

## TRANSPARENCY STATEMENT

The lead author Mohammed Mahmmoud Fadelallah Eljack affirms that this manuscript is an honest, accurate, and transparent account of the study being reported; that no important aspects of the study have been omitted; and that any discrepancies from the study as planned (and, if relevant, registered) have been explained.

## Supporting information

Supporting information.

Supporting information.

Supporting information.

## Data Availability

The data sets used and/or analyzed during the current study are available from the corresponding author on reasonable request.
