# Supplementary material for: Effects of SARS‐CoV‐2 infection and COVID‐19 pandemic on menstrual health of women: A systematic review
Source: Health Sci Rep. 2022 Oct 8;5(6):e881. doi: 10.1002/hsr2.881 (PMC9547349; doi:10.1002/hsr2.881)
Supplement: Supplementary file 2 — Supporting information. [file HSR2-5-e881-s002.docx]

**Table S1: Search strategy used in each database**

| **Database** | **Search strategy** | **Obtained articles** |
| --- | --- | --- |
| PUBMED | ("covid 19"[All Fields] OR "covid 19"[MeSH Terms] OR "covid 19 vaccines"[All Fields] OR "covid 19 vaccines"[MeSH Terms] OR "covid 19 serotherapy"[All Fields] OR "covid 19 serotherapy"[Supplementary Concept] OR "covid 19 nucleic acid testing"[All Fields] OR "covid 19 nucleic acid testing"[MeSH Terms] OR "covid 19 serological testing"[All Fields] OR "covid 19 serological testing"[MeSH Terms] OR "covid 19 testing"[All Fields] OR "covid 19 testing"[MeSH Terms] OR "sars cov 2"[All Fields] OR "sars cov 2"[MeSH Terms] OR "severe acute respiratory syndrome coronavirus 2"[All Fields] OR "ncov"[All Fields] OR "2019 ncov"[All Fields] OR (("coronavirus"[MeSH Terms] OR "coronavirus"[All Fields] OR "cov"[All Fields]) AND 2019/11/01:3000/12/31[Date - Publication]) OR ("sars cov 2"[MeSH Terms] OR "sars cov 2"[All Fields] OR "sars cov 2"[All Fields]) OR ("coronavirus"[MeSH Terms] OR "coronavirus"[All Fields] OR "coronaviruses"[All Fields])) AND ("menstrual cycle"[MeSH Terms] OR ("menstrual"[All Fields] AND "cycle"[All Fields]) OR "menstrual cycle"[All Fields] OR ("menstruation disturbances"[MeSH Terms] OR ("menstruation"[All Fields] AND "disturbances"[All Fields]) OR "menstruation disturbances"[All Fields] OR ("irregular"[All Fields] AND "menstrual"[All Fields] AND "cycle"[All Fields]) OR "irregular menstrual cycle"[All Fields]) OR (("menstrual cycle"[MeSH Terms] OR ("menstrual"[All Fields] AND "cycle"[All Fields]) OR "menstrual cycle"[All Fields]) AND ("irregular"[All Fields] OR "irregularities"[All Fields] OR "irregularity"[All Fields] OR "irregulars"[All Fields])) OR (("delay"[All Fields] OR "delayed"[All Fields] OR "delaying"[All Fields] OR "delays"[All Fields]) AND ("menstrual cycle"[MeSH Terms] OR ("menstrual"[All Fields] AND "cycle"[All Fields]) OR "menstrual cycle"[All Fields])) OR ("Early"[All Fields] AND ("menstrual cycle"[MeSH Terms] OR ("menstrual"[All Fields] AND "cycle"[All Fields]) OR "menstrual cycle"[All Fields])) OR (("prolong"[All Fields] OR "prolongation"[All Fields] OR "prolongations"[All Fields] OR "prolonged"[All Fields] OR "prolonging"[All Fields] OR "prolongs"[All Fields]) AND ("menstrual cycle"[MeSH Terms] OR ("menstrual"[All Fields] AND "cycle"[All Fields]) OR "menstrual cycle"[All Fields])) OR (("menstrual cycle"[MeSH Terms] OR ("menstrual"[All Fields] AND "cycle"[All Fields]) OR "menstrual cycle"[All Fields]) AND ("disturb"[All Fields] OR "disturbance"[All Fields] OR "disturbances"[All Fields] OR "disturbancies"[All Fields] OR "disturbed"[All Fields] OR "disturbing"[All Fields] OR "disturbs"[All Fields])) OR ("amenorrheas"[All Fields] OR "amenorrhoea"[All Fields] OR "amenorrhea"[MeSH Terms] OR "amenorrhea"[All Fields]) OR ("polymenorrhoea"[All Fields] OR "menstruation disturbances"[MeSH Terms] OR ("menstruation"[All Fields] AND "disturbances"[All Fields]) OR "menstruation disturbances"[All Fields] OR "polymenorrhea"[All Fields]) OR ("dysmenorrhea"[MeSH Terms] OR "dysmenorrhea"[All Fields] OR "dysmenorrheas"[All Fields] OR "dysmenorrhoea"[All Fields]) OR ("hypomenorrhoea"[All Fields] OR "menstruation disturbances"[MeSH Terms] OR ("menstruation"[All Fields] AND "disturbances"[All Fields]) OR "menstruation disturbances"[All Fields] OR "hypomenorrhea"[All Fields]) OR ("hypermenorrhoea"[All Fields] OR "menorrhagia"[MeSH Terms] OR "menorrhagia"[All Fields] OR "hypermenorrhea"[All Fields])) | 73 |
| COCHRANE CENTRAL | ("COVID-19" OR "SARS-CoV-2" OR "coronavirus" AND "Menstrual Cycle" OR "Irregular Menstrual Cycle" OR "Menstrual Cycle Irregularities" OR "Delayed Menstrual Cycle" OR "Early Menstrual Cycle" OR "Prolonged Menstrual Cycle" OR "Menstrual Cycle Disturbances" OR "Amenorrhea" OR "Polymenorrhea" OR "Dysmenorrhea" OR "Hypomenorrhea" OR "Hypermenorrhea") | 15312 |
| GOOGLE SCHOLAR | ("COVID-19" OR "SARS-CoV-2" OR "coronavirus" AND "Menstrual Cycle" OR "Irregular Menstrual Cycle" OR "Menstrual Cycle Irregularities" OR "Delayed Menstrual Cycle" OR "Early Menstrual Cycle" OR "Prolonged Menstrual Cycle" OR "Menstrual Cycle Disturbances" OR "Amenorrhea" OR "Polymenorrhea" OR "Dysmenorrhea" OR "Hypomenorrhea" OR "Hypermenorrhea") | 14300 |
| ERIC | (COVID-19 OR SARS-CoV-2 OR coronavirus) AND (Menstrual Cycle OR Irregular Menstrual Cycle OR Menstrual Cycle Irregularities OR Delayed Menstrual Cycle OR Early Menstrual Cycle OR Prolonged Menstrual Cycle OR Menstrual Cycle Disturbances OR Amenorrhea OR Polymenorrhea OR Dysmenorrhea OR Hypomenorrhea OR Hypermenorrhea) | 820 |
