# Supplementary material for: Effects of SARS‐CoV‐2 infection and COVID‐19 pandemic on menstrual health of women: A systematic review
Source: Health Sci Rep. 2022 Oct 8;5(6):e881. doi: 10.1002/hsr2.881 (PMC9547349; doi:10.1002/hsr2.881)
Supplement: Supplementary file 3 — Supporting information. [file HSR2-5-e881-s003.docx]

**Table S1: A detailed Newcastle-Ottawa Scale of each included cross-sectional study**

|  | Selection | | | | Comparability | Outcome | |  |
| --- | --- | --- | --- | --- | --- | --- | --- | --- |
| Study | Representativeness of the sample | Sample size | Non-respondents | Ascertainment of the exposure (risk factor) | Confounding factors controlled | Assessment of outcome | Statistical test | **Total Quality Score out of 8** |
| Aolymat et al. (2022) | 1 | 1 | 1 | 0 | 2 | 1 | 1 | **7** |
| Buran et al. (2022) | 1 | 1 | 0 | 0 | 2 | 1 | 1 | **6** |
| Schwab et al. (2021) | 1 | 1 | 0 | 0 | 2 | 1 | 1 | **6** |
| Demir et al. (2021) | 1 | 1 | 0 | 0 | 2 | 1 | 1 | **6** |
| Takmaz et al. (2021) | 1 | 1 | 0 | 0 | 2 | 1 | 1 | **6** |
| Prabowo et al. (2021) | 1 | 1 | 0 | 0 | 2 | 1 | 0 | **7** |
| Aolymat (2021) | 1 | 1 | 0 | 0 | 2 | 0 | 0 | **4** |
| Li et al (2021) | 1 | 1 | 1 | 1 | 2 | 1 | 1 | **8** |
| Mehar et al (2022) | 1 | 1 | 0 | 0 | 2 | 1 | 1 | **6** |

**Table S2: A detailed Newcastle-Ottawa Scale of each included cohort study**

|  | Selection | | | | Comparability | | Outcome | | |  |
| --- | --- | --- | --- | --- | --- | --- | --- | --- | --- | --- |
| Study | Representativeness of exposed cohort | Selection of non-exposed cohort | Ascertainment of exposure | Demonstration that the outcome of interest was present or not at the start | Study controls for age and gender | Study control for additional factors | Ascertainment of outcome | Was follow-up long enough? | Adequacy of follow-up | **Total Quality Score out of 9** |
| Ozimek et al. (2022) | 1 | 1 | 0 | 1 | 1 | 1 | 1 | 0 | 0 | **6** |
| Haile et al. (2022) | 1 | 1 | 1 | 1 | 1 | 1 | 1 | 0 | 0 | **7** |
| Nguyen et al. (2021) | 1 | 1 | 1 | 1 | 1 | 1 | 1 | 0 | 0 | **7** |
| Yuksel et al. (2020) | 1 | 1 | 1 | 1 | 1 | 1 | 1 | 0 | 0 | **7** |
| Khan MS et al (2021) | 1 | 1 | 1 | 1 | 1 | 1 | 1 | 0 | 0 | **7** |
| Ding et al (2021) | 1 | 1 | 1 | 1 | 1 | 1 | 1 | 0 | 0 | **7** |
